# Seesaw‐like movement of the chest wall

**DOI:** 10.1002/ccr3.1484

**Published:** 2018-03-12

**Authors:** Nozomi Niimi, Nobuaki Mori

**Affiliations:** ^1^ Department of General Internal Medicine National Hospital Organization Tokyo Medical Center Tokyo Japan

**Keywords:** Cardiac examination, Apical impulse, Tricuspid regurgitation

## Abstract

Examination of apical impulse is an important component of nonauscultatory cardiac examination. Seesaw‐like movements of the chest wall during the systolic phase suggest severe tricuspid regurgitation.

## Case

A 79‐year‐old woman presented with a one‐month history of pitting edema of the bilateral legs. She had undergone hepatic resection twice for a carcinoid tumor 20 years prior. Physical examination revealed inward movement of the apex impulse and simultaneous outward movement of the subxiphoid region during the systolic phase (Video S1). Transthoracic echocardiography revealed mild pulmonary hypertension and severe tricuspid regurgitation. She was diagnosed with right heart failure. The dilated right ventricle occupies the apex region in severe tricuspid regurgitation. The apex impulse is inward, and back flow can be seen at the subxiphoid region during right ventricle contraction.

## Patient Consent

We obtained the consent of the patient for the publication of the medical data. Patient privacy was fully protected, and personal information was handled such that patient could not identify.

## Authorship

NN: cared patient and wrote the manuscript. NM: contributed to critical reading of the manuscripts. All authors approved the final version of the report submitted to the journal.

## Conflict of Interest

The authors declare that they have no conflict of interest.

## Supporting information


**Video S1.** Inward movement of the apex impulse and simultaneous outward movement of the subxiphoid region during the systolic phase.

